# A deep learning-based automatic tool for measuring the lengths of linear scars: forensic applications

**DOI:** 10.1093/fsr/owad010

**Published:** 2023-03-27

**Authors:** Jian Zhou, Zhilu Zhou, Xinjian Chen, Fei Shi, Wentao Xia

**Affiliations:** School of Electronics and Information Engineering, Soochow University, Suzhou, China; Shanghai Key Laboratory of Forensic Medicine, Shanghai Forensic Service Platform, Academy of Forensic Science, Ministry of Justice, Shanghai, China; Department of Forensic Medicine, Guizhou Medical University, Guiyang, China; School of Electronics and Information Engineering, Soochow University, Suzhou, China; School of Electronics and Information Engineering, Soochow University, Suzhou, China; Fariver Innovation Technologies Co, Ltd, Suzhou, China; Shanghai Key Laboratory of Forensic Medicine, Shanghai Forensic Service Platform, Academy of Forensic Science, Ministry of Justice, Shanghai, China

**Keywords:** photogrammetry technology, multiview stereo, deep learning, structure from motion, image segmentation

## Abstract

It is important to measure scars in forensic and clinical medicine. In practice, scars are mostly manually measured, and the results are diverse and influenced by various subjective factors. With the development of digital image technology and artificial intelligence, noncontact and automatic photogrammetry has been gradually used in some practical applications. In this article, we propose an automatic method for measuring the length of linear scars based on multiview stereo and deep learning, which combines the 3D reconstruction algorithm of structure from motion and the image segmentation algorithm based on a convolutional neural network. With a few pictures taken by a smart phone, automatic segmentation and measurement of scars can be realized. The reliability of the measurement was first demonstrated through simulation experiments on five artificial scars, giving errors of length <5%. Then, experiment results on 30 clinical scar samples showed that our measurements were in high agreement with manual measurements, with an average error of 3.69%. Our study demonstrates that the application of photogrammetry in scar measurement is effective and that the deep learning technique can realize the automation of scar measurement with high accuracy.

## Introduction

Skin scar means that skin tissue is completely damaged and then fibrillar connective tissue is formed during healing process [1]. In forensic science, it is generally acknowledged that injury time and vulnerant can be inferred according to the linear scars’ colour, length, and shrinkage. It has been found that the scar caused by different wound tools has different morphological characteristics [2]. For instance, the scar’s shrinking rate caused by blunt instrument injury is higher than that caused by sharp instrument injury [3]. Therefore, scar measurement is often required in forensics to assist crime investigation and to protect the legitimate rights and interests of victims [4, 5]. Areal or volumetric measurements can also be used to assess the treatment outcomes in clinical medicine [5–7].

Traditional scar measurements with ruler, palm, film tracing method, etc., still continue to be commonly used methods, which take longer time and need contact of bodies during examinations [8]. Moreover, when meeting uneven parts, such as ear and nose, accurate results will be difficult to obtain by these measurements. In addition, it has been proved that scar normally stabilizes in 3 months, but its consistent changing cannot be excluded, such as scar atrophy. Therefore, the evidence from traditional methods has poor fidelity and storability [2]. The new measurement method, such as structured light 3D scanning technology, has been developed and can extract the isolated information of skin scars, with accurate and reliable results [9]. However, the method has some disadvantages. For instance, the instrument is not portable, has motion artefact, and the scanning light is harsh. Therefore, it is necessary to find a noncontact, portable, quick imaging, and highly accurate method for scar measurement.

Photogrammetry is the science and technology of obtaining reliable information about physical objects and the environment through the process of recording, measuring, and interpreting photographic images and patterns of electromagnetic radiant imagery and other phenomena [10]. Photogrammetry is often used to acquire 3D data and measure nonrigid objects. It involves the process of taking multiple perspective images of the object to be measured and then reconstructing a 3D model. In forensics, photogrammetry can be applied in measuring crime scene, height of human, specific organ tissues, skin wounds, and other injuries [11–16]. The accuracy of photogrammetry depends on the clarity and quantity of images acquired, the distortion of the camera, and size of the subject within the image.

In recent years, artificial intelligence (AI) has developed rapidly and is widely used in various fields. The application of deep learning in the field of computer vision has gradually matured [17, 18]. Especially, convolutional neural networks (CNNs) have revolutionized tasks such as image recognition, classification, and semantic segmentation [19]. At the same time, CNNs has achieved great success in the field of segmentation of medical images (MRI, CT, X-ray, etc.) and auxiliary diagnosis due to its excellent feature expression capabilities, and with the segmentation of medical images, clinicians can quantitatively analyse the pathological area [20]. The present research attempts to use AI and photogrammetry to achieve automatic segmentation and measurement of linear scars.

In our study, scars are adhered to the surfaces of human skin, which are not simple planes but curved surfaces in 3D space. Therefore, the measurement scheme based on 2D vision is not accurate. In order to use the existing technology to measure the length of linear scars and to solve practical problems in the forensic medicine, we propose an automatic segmentation and measurement method for linear scars based on multiview 3D reconstruction and CNNs. First, 3D reconstruction technology based on multiview images is applied to restore the 3D information of the scar. Then, a 2D CNN is used to automatically segment the scar in multiview images. Finally, the results of these two techniques are combined to generate point cloud data, and the length of linear scars is calculated based on point cloud processing. As far as we know, this is the first attempt for noncontact, automatic, and 3D measurement of scars.

The rest of the paper is organized as follows: in the Related works section, we introduce some previous work on photogrammetry, scar measurement, and wound measurement. In the Materials and methods section, the overall measurement framework, each step of the proposed method, evaluation metrics, experimental data, and implementation details will be described and explained in detail. Results of image segmentation and length measurement are given in the Results section. In the end, we give some discussions and conclude our entire work.

## Related works

There is extensive application of photogrammetry in the field of forensic medicine. Zou et al. [12] used traditional measurement and single-camera photogrammetry to measure 19 skulls, respectively, and analysed the differences between the two measurement methods and the intragroup differences between groups of different focal lengths with the same photographic equipment. Flies et al. [13] documented 33 cadaveric skin lesions using photographs and video recordings, and lesion analysis were performed by manual and automatic point measurements, respectively. They concluded that the differences between the manual point and automatic point measurements were very small. Donato et al. [14] generated 3D volumes of skulls to compare the photogrammetry versus the CT scan. Their experiment results indicated that the measurements taken on the photogrammetry-based skull tend to slightly overestimate compared with those taken on the CT-based skull. Koller et al. [15] used 3D photogrammetry to overcome the shortcomings of spatial information loss of 2D images and achieved accurate measurement of wound size. Lee et al. [16] proposed a photogrammetry-based method for criminal height measurement using surveillance camera images, and it was found that the motion of a person can lead to measurement errors.

However, at present, there are relatively few studies on the measurement of scars. Gao [8] proposed to place thin copper wire close to the body surface and mark the length. Then, the thin copper wire is straightened and measured with a ruler which represents the scar length. Min and Zhang [21] directly used a 3D scanner to scan the scar area to obtain point cloud data. Then, they extracted the trend line of the scar based on the point cloud data. Finally, the length of the scar is solved by calculating the trend line. Fu et al. [9] proposed a method to measure the length of linear scars, based on structured light technology, by which point cloud data were obtained and processed. Then, they manually extracted the scar edge and calculated the scar length [9]. Taylor et al. [7] used a 3D laser imaging device and interactive image processing to measure the volume of keloid scars. These methods require special imaging devices and/or involve manual intervention.

Similar research has been carried out for wound measurement. Treuillet et al. [22] proposed a 3D assessment tool for skin wounds. They restored a 3D model of the wound by taking two images from different angles and calculated wound volume based on triangulation. Pavlovčič and JezeršEk [23] proposed to use a handheld measurement system based on triangulation and structured light technology to measure wound size. Filko et al. [24] explored 3D detection, segmentation, reconstruction, and measurement of wounds based on the images taken by an RGB-D camera. Some of these methods achieved noncontact, automatic, and 3D measurement of wounds. However, the image features of scars are very different from those of wounds, and they are more difficult to identify from normal skin. Therefore, more powerful image processing methods need to be applied.

## Materials and methods

### Method overview

**Figure 1:**
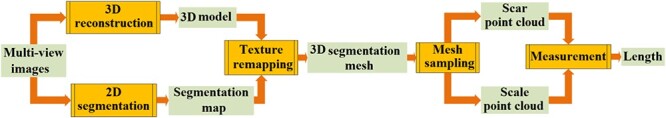
Flowchart of the automatic scar measurement method.

The overall framework of automatic measurement is shown in Figure 1. Multiview images are taken, with a scale placed beside the scar being measured. In this study, we used a sticker scale attached to the normal skin near the scar, with a round shape whose radius is 0.5 cm and whose colour is blue or green. The multiview images are, respectively, passed through the 3D reconstruction module and the 2D image segmentation module. The segmentation mesh of the 3D model can be obtained by remapping the 2D segmentation maps to the 3D model. The point clouds of the scar and scale can then be extracted and measured, respectively, by point cloud processing. Finally, the true scar length is obtained based on the proportion of sizes between the scar and the scale.

### 3D reconstruction

We use the structure from motion (SfM) algorithm [25] to achieve 3D scene reconstruction from multiview-images taken by a digital camera or smart phone. The first step is feature extraction. Feature points with scale, rotation, and illumination invariance in each image are extracted. The second step is feature matching. Feature points are matched based on the Euclidean distance to generate image matching pairs. The third step is sparse reconstruction. Sparse point cloud in the scene is reconstructed. The fourth step is dense reconstruction. Dense point cloud in the scene is reconstructed. The final step is mesh reconstruction and texture mapping. Then, a complete 3D model is reconstructed based on the multiview method.

### Image segmentation

**Figure 2:**
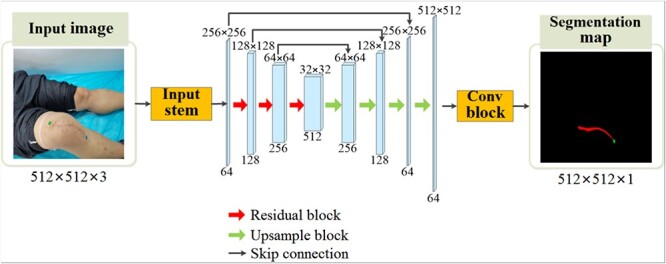
Image segmentation framework based on convolutional neural network; the red area in the segmentation map represents the linear scar, and the green area represents the scale.

The deep network we use has an encoder–decoder structure, which is commonly used in the field of image segmentation. In order to get more representative feature maps, we employ a pretrained ResNet34 [26] as the encoder. ResNet, the deep residual network, is a milestone in deep learning-based image processing. The residue structure can effectively eliminate the degeneration problem caused by the increase in the number of convolutional layers and can make the network easier to train. In addition, the pretrained models of these classic networks based on the large-scale ImageNet database can be used to promote other tasks [26]. As shown in Figure 2, the encoder consists of four feature extraction layers of ResNet34 [26], and the decoder consists of four consecutive convolutional layers and upsampling operations. The skip connection between the encoder and the decoder is used to combine the feature maps in the deep layers of decoder representing semantic information and the ones in the shallow layers of encoder containing detailed information. We removed the maxpooling layer in the Input stem block to improve the segmentation of slender objects.

In order to improve the performance of our model, we use a joint loss function in model training, including Dice loss, *L*_Dice_, and binary crossentropy (BCE), loss *L*_BCE_, defined as follows:


(1) \begin{equation*} {L}_{\mathrm{Dice}}=1-\frac{2\times \sum_i^N{p}_i{g}_i+\varepsilon }{\sum_i^N{p_i}^2+\sum_i^N{g_i}^2+\varepsilon },\qquad\qquad\qquad \end{equation*}

(2) \begin{equation*} {L}_{\mathrm{BCE}}=-\frac{1}{N}\sum_i^N{g}_i\log \left({p}_i\right)+\left(1-{g}_i\right)\log \left(1-{p}_i\right), \end{equation*}

where ${p}_i\in \left[0,1\right]$ denotes the predicted probability for the scar or scale region, and ${g}_i\in \left\{0,1\right\}$ denotes the ground truth label for scar or scale region.


(3) \begin{equation*} {L}_{\mathrm{total}}={L}_{\mathrm{Dice}}+{L}_{\mathrm{BCE}}. \end{equation*}

### Texture remapping and point cloud segmentation

The 3D reconstruction recovers the scene information, and the 2D image segmentation obtains the position information of the scar and the scale. Based on the 2D to 3D mapping obtained in the reconstruction step, we can map the 2D segmentation map to 3D with the same correspondence. Then, the 3D segmentation mesh can be converted into point clouds through mesh sampling operation. Colour thresholding is used to extract the point clouds corresponding to scar and scale from the black background.

### Length measurement

As the actual radius of the scale is 0.5 cm, as long as the pixel-space radius of the round scale, $R$, and the linear scar length, ${L}_{\mathrm{pc}}$, are solved, the actual length of the linear scar can be calculated as


(4) \begin{equation*} {L}_{\mathrm{scar}}=\frac{L_{\mathrm{pc}}}{2R}\ \left(\mathrm{cm}\right). \end{equation*}

The median of distance values calculated between the centre and all edge points of the scale are taken as the radius $R$. Since sampling of the point cloud is random, we take the average of multiple measurements.

**Table 1:** Results of comparative experiments.

Method	DSC	Sen	Spe
U-Net [29]	0.8002	0.8619	0.9990
Attention U-Net [30]	0.7957	0.8486	0.9991
FCN_Res50 [31]	0.8303	0.8725	0.9990
DeepLabV3_ Res50 [32]	0.8389	0.9018	0.9990
CE-Net [33]	0.8463	0.8900	0.9991
The present study	0.8660	0.9029	0.9993

DSC, Dice similarity coefficient; Sen, sensitivity; Spe, specificity.

For calculating the length of a linear scar, we propose a point cloud skeleton extraction algorithm based on neighbourhood mean. For the point cloud corresponding to the linear scar, after removal of outliers, we define its main direction according to the largest span in the *X*, *Y*, or *Z* directions and reset this direction as the *X* axis, without loss of generality. We set the resolution of the skeleton as 1 point/mm in the main direction. Therefore, the total range of points in the main direction $\left[0,{X}_{\mathrm{range}}\right]$ is divided into nonoverlapping windows $\left\{{w}_{m,}\ m=1,\cdots, M\right\}$ with size $R/10$, where $M=\left[{X}_{\mathrm{range}}/R\times 10\right]$. Then, the mean coordinate of points falling in to the same window is calculated as the position of a skeleton point.


(5) \begin{equation*} {\displaystyle \begin{array}{c}{P}_m^{\mathrm{skl}}=\mathrm{mean}\left({P}_i\right),{x}_i\in{w}_m\\{}{P}_i=\left[{x}_i,{y}_i,{z}_i\right]\end{array}}\!\!\!\!\,. \end{equation*}

### Evaluation metrics

In order to objectively evaluate the segmentation performance of our model, three common evaluation metrics were used, including Dice similarity coefficient (DSC), sensitivity (Sen), and specificity (Spe), calculated as follows:


(6) \begin{equation*} \mathrm{DSC}=\frac{2\times \mathrm{TP}}{2\times \mathrm{TP}+\mathrm{FP}+\mathrm{FN}},\qquad \end{equation*}

(7) \begin{equation*} \mathrm{Sen}=\frac{\mathrm{TP}}{\mathrm{TP}+\mathrm{FN}}, \qquad\qquad\qquad \ \ \, \end{equation*}

(8) \begin{equation*} \mathrm{Spe}=\frac{\mathrm{TN}}{\mathrm{TN}+\mathrm{FP}}, \ \, \qquad\qquad\qquad \end{equation*}

where TP, TN, FP and FN represent the number of true positive, true negative, false positive, and false negative predictions, respectively.

To evaluate the accuracy of scar length measurement, relative error (RE) was used, which was calculated as follows:


(9) \begin{equation*} \mathrm{RE}=\frac{\left|{X}_a-{X}_r\right|}{X_r}\times 100\%, \end{equation*}

where ${X}_a$ represents automatic measurement and ${X}_r$ represents reference value.

### Materials

To verify the feasibility of measurement in the reconstructed 3D model, some simulation experiments were performed. In the experiments, we drew five “artificial scars” with different curvatures and lengths using the AutoCAD software and printed the image at 1:1 ratio. The paper with the artificial scar was attached to a curved surface. Four multiview images were taken for each artificial scar. The true length of these scars was calculated directly in the AutoCAD software. In order to independently verify the accuracy in length measurement, the scar and scale were manually annotated.

Our clinical dataset was collected from the Academy of Forensic Science, Ministry of Justice, P. R. China with written approval for usage in this study. Each scar sample had the result of manual measurement made by a forensic expert. All the scar images were taken from multiple angles with a smart phone (HUAWEI Nova 7; HuaweiTech, Shenzhen, China) with a single camera. The subject was kept still and the smart phone was moved around at the time of data collection. It was also made sure that the entire scar appeared in the field of view and was about in the centre of the image. A total of 778 images were collected from 130 scar samples. Each sample contained 4–10 multi-view images. As required by CNN model developing, we divided the dataset as follows: 80 scars containing 485 pictures for training, 20 scars containing 124 pictures for segmentation model validation, and 30 scars contain 169 pictures for testing.

### Implementation details

The experiments were carried out on a PC with Intel^®^ Core™ i7-9700 CPU @ 3.00GHz, 16GB RAM, and NVIDIA GeForce RTX 2060 SUPER GPU with 8G memory. The 3D reconstruction algorithm was implemented based on the C++ open source library of Colmap [27] and OpenMVS [28]. The image segmentation algorithm was implemented on the Pytorch deep learning platform, with GPU acceleration.

In order to balance between the accuracy and efficiency of 3D reconstruction, the image was downsampled by a rate of 4 before the mesh was refined. The minimum resolution for image after rescaling is 960. The number of views used for depth map estimation is set to 3. The sampling density per square unit of mesh is 1 000.

For image segmentation, the input image of the segmentation network was downsampled to 512 × 512. Data augmentation was used in the training process, such as horizontal flipping, vertical flipping, and rotations from −30° to 30°. The number of epochs for network training was set to 200 and the batch size was set to 2. The “poly” learning rate policy was used, where $\mathrm{lr}={\mathrm{lr}}_{\mathrm{base}}\times{\left(1-\mathrm{iter}/\mathrm{total}\_\mathrm{iter}\right)}^{\mathrm{power}}$, the basic learning rate ${\mathrm{lr}}_{\mathrm{base}}$ was set to 0.01, and $\mathrm{power}$ was set to 0.9. The optimizer was stochastic gradient descent in which momentum and weight decay were set to 0.9 and 0.0001, respectively.

**Figure 3:**
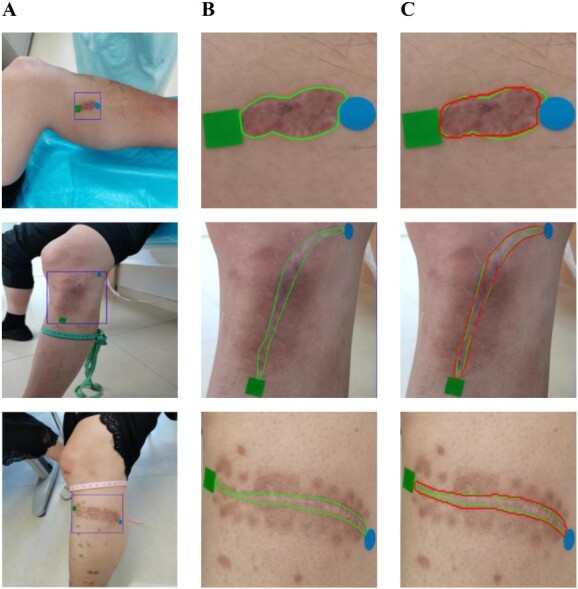
Image segmentation results; (A) original image, (B) ground truth, and (C) the present study (red contours) compared with ground truth (green contours).

**Figure 4:**
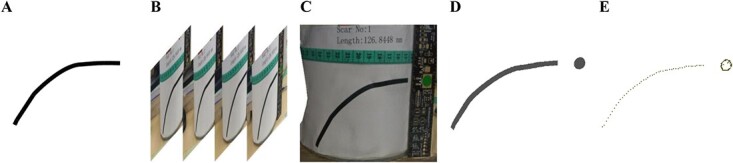
Simulation experiments; (A) artificial scar, (B) multiview images, (C) reconstructed model, (D) point clouds of scar and scale, and (E) skeleton of scar and edge of scale.

**Table 2:** Results of the simulation experiments.

No.	Number of views	CAD measurement (cm)	Our measurement (cm)	RE (%)
Scar 1	4	12.68	12.35	2.60
Scar 2	4	6.80	6.58	3.23
Scar 3	4	1.41	1.36	3.54
Scar 4	4	10.42	10.03	4.70
Scar 5	4	20.01	19.53	2.39

RE, relative error; CAD: computer aided design.

**Figure 5:**
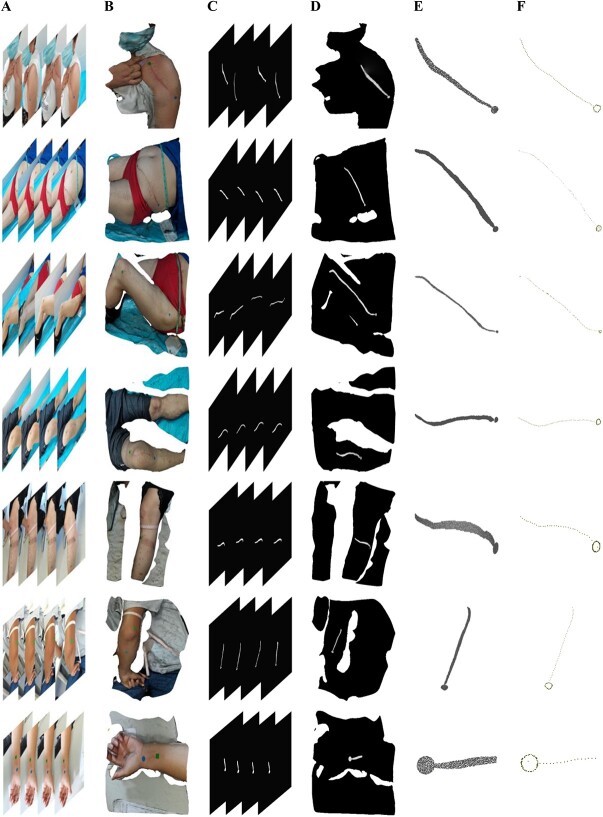
Clinical experiments; (A) multiview clinical images, (B) reconstructed models, (C) 2D segmentation maps, (D) segmentation results remapped to 3D model, (E) point clouds of scars and scales, and (F) skeletons of scars and edges of scales.

## Results

### Image segmentation

The results of 2D scar segmentation are shown in Table 1. We compared our method with other excellent CNN based methods, including U-Net [29], attention U-Net [30], fully convolutional networks [31], DeepLabV3 [32], and CE-Net [33]. The DSC of our method reaches 0.8660. This proves the effectiveness of our model. Some visualization of segmentation results is shown in Figure 3. Note that small discrepancies in contours may not affect the accuracy of the following length measurement.

### Simulation experiment

One example of simulation experiment is shown in Figure 4, and the results of measurement are shown in Table 2. We present the artificial scar, the multiview images used for reconstruction, the reconstructed 3D model, the point cloud of the scar and scale, and the skeleton of the scar and the edge of the scale, respectively. It can be seen from the results that compared with the true length, our measurements give errors <5%.

### Clinical experiment

We applied our method to 30 sets of clinical data. Like the simulation experiment, we gave some visualized results, including multiview images used for reconstruction, the reconstructed 3D model, the segmentation maps of the multiview images, the segmentation mesh of the 3D model, the point cloud of the scar and the scale, and the skeleton of the scar and the edge of the scale. The results show scars of different lengths in different areas of the human body, including thighs, calves, abdomen, shoulders, wrists, elbows, ankles, knees, etc. (Figure 5).

**Figure 6:**
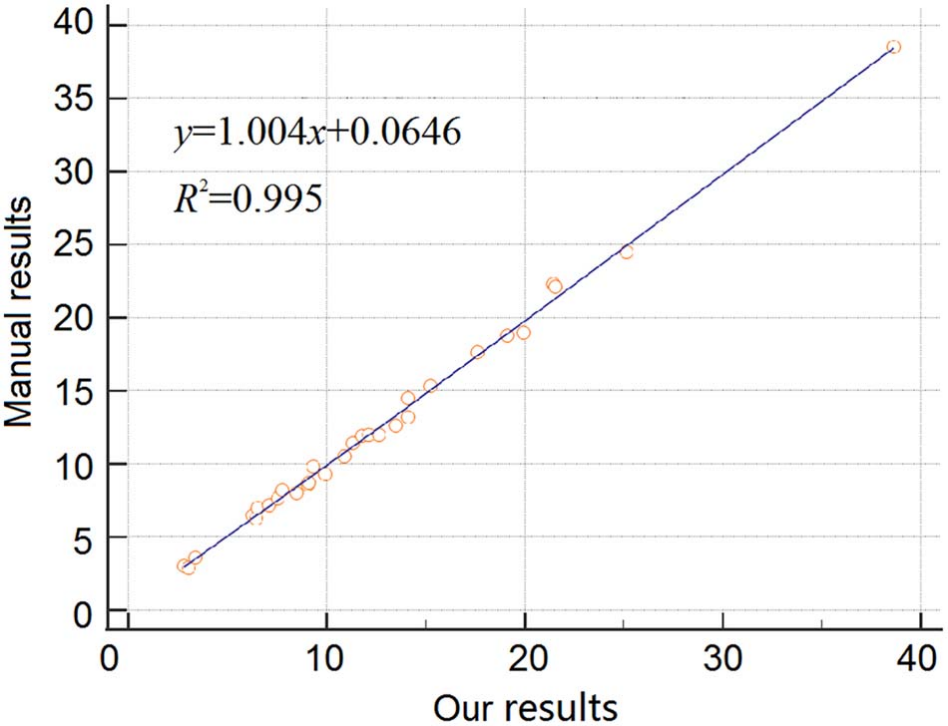
Correlation analysis diagram.

**Figure 7:**
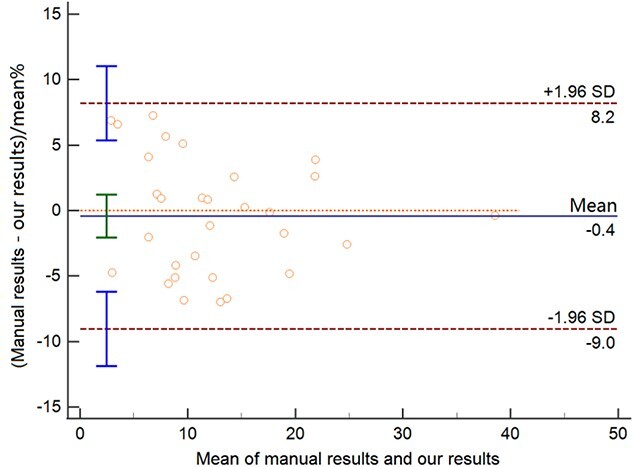
Bland–Altman diagram; SD: standard deviation.

The measurement results of 30 sets of clinical data are listed in Table 3. We compared our results with manual measurements. The average error between our result and manual result is 3.69%. The maximum error value is 7.22%, and the minimum error value is 0.11%. The correlation analysis diagram and the Bland–Altman diagram are shown in Figures 6 and 7, respectively, which indicate our results have strong correlation with manual results.

**Table 3:** The results of clinical experiments.

No.	Time cost (s)		Number of views	Manual (cm)	Ours (cm)	RE (%)
	Reconstruction	Measurement				
Scar 1	154.73	24.67	6	22.1	21.53	2.57
Scar 2	95.21	24.05	5	9.3	9.96	7.09
Scar 3	119.91	24.01	7	7.6	7.53	0.92
Scar 4	127.72	25.98	6	9.8	9.31	5.00
Scar 5	105.64	32.42	5	11.9	11.80	0.84
Scar 6	100.80	7.46	4	12.6	13.51	7.22
Scar 7	112.53	35.98	5	22.3	21.45	3.81
Scar 8	104.45	23.70	5	38.5	38.64	0.36
Scar 9	145.36	26.38	5	2.9	3.04	4.82
Scar 10	125.76	26.18	5	10.5	10.87	3.52
Scar 11	89.87	24.31	6	14.5	14.13	2.55
Scar 12	88.52	25.50	5	18.8	19.13	1.75
Scar 13	126.21	24.74	6	24.5	25.14	2.61
Scar 14	109.94	25.84	5	3.6	3.37	6.38
Scar 15	89.23	45.99	4	3.0	2.80	6.66
Scar 16	94.25	30.09	5	8.2	7.75	5.48
Scar 17	101.95	27.13	5	11.4	11.29	0.96
Scar 18	121.78	26.47	6	12.0	12.14	1.16
Scar 19	134.33	25.79	6	13.2	14.12	6.96
Scar 20	86.10	59.62	4	15.3	15.26	0.26
Scar 21	106.95	34.18	7	6.3	6.43	2.06
Scar 22	105.34	26.17	7	7.0	6.51	7.00
Scar 23	102.52	26.90	6	8.7	9.07	4.25
Scar 24	92.23	27.13	5	8.0	8.46	5.75
Scar 25	87.57	26.44	5	12.0	12.63	5.25
Scar 26	108.48	30.41	5	17.6	17.62	0.11
Scar 27	151.86	24.11	9	19.0	19.94	4.95
Scar 28	95.04	27.28	5	6.5	6.24	4.00
Scar 29	126.80	24.48	7	7.2	7.11	1.25
Scar 30	142.03	31.14	8	8.6	9.05	5.23
Mean ± Std	111.77 ± 19.95	28.15 ± 8.38	/	/	/	3.69 ± 2.35

RE, relative error; Std, standard deviation.

## Discussion

We propose an automatic, noncontact, and objective method for scar length measurements in 3D space. We combine the 3D reconstruction based on the SfM algorithm with the image segmentation algorithm based on deep learning to complete the segmentation, which transforms the problem of complex 3D model segmentation into the easy problem of 2D image segmentation. It is demonstrated that, based on

multiview images captured by an ordinary smart phone, the reconstructed 3D model is visually realistic, clearly reflecting the shape, location, and curvature of the scar. The powerful data-driven deep learning method is adopted to automatically delineate the scar area. The DSC of our segmentation model reached 0.866, and it outperforms some state-of-the-art deep neural networks. Then, we proposed a linear scar measurement method on point cloud data based on skeleton extraction. Simulation experiments show that the 3D measurement based on manual segmentation gives errors <5% compared with ground truth. Combining automatic segmentation and point cloud measurement, a fully automatic 3D scar length measurement framework is designed. For the 30 sets of clinical data, the mean relative difference between our calculated results and manual measurement results is 3.69%. The correlation between manual and automatic measurements is also high.

Traditional manual measurement can be affected by subjective factors and requires touching of the skin. The methods based on 2D images can only measure the projection of scars on a plane, which greatly reduces the accuracy, especially when the scars lie on a surface with large curvatures. Most of the previous research on linear scar measurement tried to deal with the above problems, but they still involved more or less manual interventions. Gao [8] used thin copper wire instead of ruler to measure scars so that the scar shape can be better fitted. However, this method requires contact, and subjective factors still cannot be avoided during manual fitting, such as the judgement of scar curvature and thickness. Min and Zhang [21] used a 3D scanner to collect point cloud data of the scar. Although they used an automatic algorithm to calculate the scar length, they ignored the error and trouble caused by manual extraction of the scar area. Their method was validated on only three sets of data. Fu et al. [9] acquired scar data using structured light, which required expensive image equipment. Moreover, they used a type of general-purpose software to interactively extract the scar area, and the segmentation subjected to the interference of backgrounds and noise. Taylor et al. [7] used a special device with laser scanning to capture the 3D scar data, where strict imaging setting, including shooting distance and lighting condition, is required. Multiple steps of interactive image processing are needed to obtain the measurement.

By contrast, the requirement of data acquisition in our method is low. Any digital camera or smart phone can be used. There are no strict requirements of shooting angle and distance, and the imaging operation is easy and noncontact. The measurement is fully automatic and thus avoids errors caused by subjective factors.

As can be seen in Figure 5, we have attached two scales near the scar to be measured. The scale colour is chosen to be blue or green, making them different from the colour of skin and scar so that they are easy to segment. One is a square with a side length of 1 cm and the other is a circle with a radius of 0.5 cm. In our study, we choose the circular scale because multiple radii can be solved and the average value can be calculated to reduce the error. The deformation of the scale can cause measurement errors. In later practice, a rigid scale such as a coin can be used.

There is still some room for improvement in our method. First, the efficiency of 3D reconstruction needs to be enhanced to facilitate clinical usage. As can be seen from the time cost in Table 3, the most time-consuming part of the whole process is 3D reconstruction. The time cost depends not only on the number of pictures but also on the image quality, resolution, and reconstruction parameters. To cope with the problem, state-of-the-art deep learning-based methods, usually with a short inference time, can be applied for feature point matching and dense reconstruction. Secondly, the automatic segmentation method can be further investigated. Modules that help better extract the colour, texture, and context information can be integrated to the network. On the other hand, instead of segmentation independently in 2D images, the context information among multiview images can be exploited. Finally, according to the nature of deep learning, if more training data are collected, the segmentation performance is expected to be further improved.

In our experiment, there is only one measurement target in each image. In the future, we will try to segment and analyse multiple scars at the same time when there are multiple scars in the same body part, which can improve the measurement efficiency. In addition, our method can be generalized to measure various types of scars and to obtain more quantitative indices such as scar area, which are harder to measure manually. The application prospect of our method is very broad. A smart phone-based scar measurement application can be further developed based on the proposed framework, and it has the potential to be applied to telemedicine. The method can also be extended to skin wound measurement and can be used in clinical medicine for recording and tracking the wound-healing process.

## Conclusion

Measurement and analysis of scars are important in forensic and clinical medicine. In this article, we proposed a deep learning-based automatic tool for measuring the length of linear scars. An average error of 3.69% in scar length measurement was achieved on clinically obtained test data. This study demonstrates that the application of photogrammetry in scar measurement is effective. The proposed method is promising in providing convenient, objective, accurate, and low-cost measurement of linear scars and can help save the workloads of forensic clinicians. It lays the foundation for further research of measurement for different degrees and types of scars.

## Authors’ contributions

Jian Zhou and Zhilu Zhou conceived the idea for the article, performed the literature search, collected the data and analysis, and drafted the figures and manuscript. Xinjian Chen, Fei Shi, and Wentao Xia funded this work, and all of them critically revised the work. All authors contributed to and revised the final version of the manuscript.

## Compliance with ethical standards

This work involved human subjects, and all of these subjects has signed the informed consent. This study has been reviewed by the Ethics Committee of the Academy of Forensic Sciences and meets the requirements of the Measures for the Ethics reviewed of Biomedical Research Involving People (2016), and the study was agreed to carry out as planned.

## Disclosure statement

None declared.

## Funding

Gradation of Personal Injury-caused Disability, Standards project, Ministry of Justice, P.R.C [grant number SF20181312].
